# Epigenetic profile in lipopolysaccharide-stimulated macrophages

**DOI:** 10.1186/cc12987

**Published:** 2013-11-05

**Authors:** Ester Correia Sarmento Rios, Thais Martins Lima Salgado, Francisco Garcia Soriano

**Affiliations:** 1 Department of Emergency Medicine, University of São Paulo Medical School, Brazil

## Background

Sepsis remains a clinical challenge for the ICUs. However, it is known that the tolerance mechanism using low doses of lipopolysaccharide (LPS) reduces the expression of proinflammatory genes and involves epigenetic regulation. The chromatin openness is regulated by histone acetyltransferases (HATs) and these enzymes could be modulated by nitric oxide (NO) interaction. In the present work, we demonstrate the pathway of tolerance to LPS from HAT activity and level of histone openness to production of cytokines as well as the influence of NO inhibition.

## Materials and methods

THP1 differentiated into macrophages (with 2.5 nM PMA treatment) were cultivated in RPMI medium (Control group), submitted to tolerance (500 ng/ml LPS 24 hours before challenge with 1,000 ng/ml LPS - Tolerant group) and challenge (1,000 ng/ml LPS - D group) during 24 hours. NO production was inhibited by addition of 100 μM LNAME. The HAT activity and cytokine production (IL-6) were measured with biochemistry kits. Histone acetylated H3 and H4 were analyzed by western blotting.

## Results

Tolerance reduced HAT activity compared with the group directly challenged (*P *< 0.05). Acetylated H4 was maintained at basal levels in the tolerant group and increased in the D group (*P *< 0.05). However, the tolerance increases the acetylation of histone H3 in a NO-dependent response. Similarly, the IL-6 release was reduced by induction of tolerance (*P *< 0.05 vs. D group). However, this effect was abolished by inhibition of NO production.

## Conclusions

The induction of tolerance diminishes HAT activity and cytokine production. The tolerance triggers a complex epigenetic modulation dependent of NO.

